# Evaluation of a commercial deformable image registration algorithm for dual‐energy CT processing

**DOI:** 10.1002/acm2.12987

**Published:** 2020-07-25

**Authors:** Jessie Y. Huang, Michael J. Lawless, Charles K. Matrosic, Lianna D. Di Maso, Jessica R. Miller

**Affiliations:** ^1^ Department of Human Oncology University of Wisconsin‐Madison Madison WI USA; ^2^ Department of Medical Physics University of Wisconsin‐Madison Madison WI USA

**Keywords:** deformable image registration, dual‐energy CT, quality assurance, TG‐132

## Abstract

**Purpose:**

Several dual‐energy computed tomography (DECT) techniques require a deformable image registration to correct for motion between the acquisition of low and high energy data. However, current DECT software does not provide tools to assess registration accuracy or allow the user to export deformed images, presenting a unique challenge for image registration quality assurance (QA). This work presents a methodology to evaluate the accuracy of DECT deformable registration and to quantify the impact of registration errors on end‐product images.

**Methods:**

The deformable algorithm implemented in Siemen Healthineers's Syngo was evaluated using a deformable abdomen phantom and a rigid phantom to mimic sliding motion in the thorax. Both phantoms were imaged using sequential 80 and 140 kVp scans with motion applied between the two scans. Since Syngo does not allow the export of the deformed images, this study focused on quantifying the accuracy of various end‐product, dual‐energy images resulting from processing of deformed images.

**Results:**

The Syngo algorithm performed well for the abdomen phantom with a mean registration error of 0.4 mm for landmark analysis, Dice similarity coefficients (DSCs) > 0.90 for five organs contoured, and mean iodine concentrations within 0.2 mg/mL of values measured on static images. For rigid sliding motion, the algorithm performed poorer and resulted in noticeable registration errors toward the superior and inferior scan extents and DSCs as low as 0.41 for iodine rods imaged in the phantom. Additionally, local iodine concentration errors in areas of misregistration exceeded 3 mg/mL.

**Conclusions:**

This work represents the first methodology for DECT image registration QA using commercial software. Our data support the clinical use of the Syngo algorithm for abdominal sites with limited motion (i.e., pancreas and liver). However, dual‐energy images generated with this algorithm should be used with caution for quantitative measurements in areas with sliding motion.

## 1 INTRODUCTION

Dual‐energy computed tomography (DECT) imaging uses attenuation information acquired with two different energy spectra; the high and low energy data, along with the known attenuation properties of basis materials, can be processed in order to obtain quantitative material information (e.g., iodine and water material images).^1^ DECT imaging has many applications in both diagnostic imaging and radiation therapy, including material differentiation, contrast enhancement,^2^ virtual noncontrast^3^ and virtual monoenergetic imaging, perfusion imaging,^4^ and metal artifact reduction.^5^

One challenge with dual‐energy imaging is the presence of motion in between the acquisition of the high energy and low energy data. If left uncorrected, this motion can cause artifacts and inaccurate values in the resulting dual‐energy images. There are several DECT acquisition techniques currently available with varying sensitivities to patient motion. The longer the temporal separation between the acquisition of the low and high energy data, the more sensitive the DECT technique is to patient motion. The sequential scan technique, in which a low energy CT scan is followed by a high energy CT scan, is the most susceptible to motion. Although less susceptible to motion than the sequential scan technique, patient motion can still impact images for other DECT techniques such as fast kVp switching, dual‐source DECT, and DECT with a split‐filter (TwinBeam).

Image registration can be used to correct motion between the high and low energy image acquisitions. This is the approach taken by the Siemens Healthineers Syngo image viewing and processing software, which deforms the low energy image to the high energy image. Syngo uses a deformable image registration that was designed specifically for liver imaging; the algorithm uses a constrained fluid‐based model that assumes that there is no change in volume of the organ and performs regularization via Gaussian smoothing.^6^ In the Syngo software, this algorithm is implemented for sequential scans and split‐filter acquisition techniques, and is automatically applied to DECT images of all anatomical sites for a number of applications including the creation of virtual monoenergetic images and iodine images. Thus, in order to be successful, this deformable image registration algorithm needs to be able to accurately account for various types of motion encountered with DECT imaging, including rigid sliding motion and organ deformation due to respiration.

The American Association of Physicists in Medicine's Task Group 132 provides recommendations for validation and quality assurance testing of image registration algorithms used for radiation therapy.^7^ TG‐132 outlines several metrics to quantify registration accuracy including the distance between landmark points on the two images sets, the Dice similarity coefficient (DSC) to quantify contour agreement, and the Jacobian determinant to quantify local volume changes due to the registration; the task group also outlines tolerance levels for these quantitative tests. Although deformable image registration algorithms are routinely used for dual‐energy CT image processing, little work has been done to evaluate the accuracy of these deformed images and the impact of image registration on the resulting DECT images. Leng et al. compared the success of the sequential scan technique, with deformable image registration applied, to the dual‐source technique for distinguishing between uric acid and nonuric acid kidney stones. Although Leng et al. compared how accurately the stones were classified in comparison to the dual‐source technique and also visually evaluated how the deformable registration algorithm improved the appearance of images, they did not perform any quantitative analysis of image registration accuracy.^8^ Skornitze et al. evaluated rigid and deformable registration algorithms for DECT perfusion imaging using qualitative radiologist ratings and a quantitative evaluation of perfusion model fitting.^9^ Outside of dual‐energy imaging, validation of rigid and deformable algorithms for motion correction in CT perfusion studies of the lung and liver has been performed.^10^, ^11^ However, to the authors' knowledge, no work has been published that applies the metrics of registration accuracy evaluation outlined in TG‐132 to algorithms in dual‐energy CT processing; nor have any studies quantified the impact of registration errors on the resulting DECT images. Applying the TG‐132 recommendations to the Syngo deformable registration algorithm is challenging because the software does not have any evaluation tools other than the ability to blend the images, nor does it have the ability to export the deformed image set or the deformation vector field. This work proposes a method to carry out a TG‐132 evaluation of registration accuracy in spite of these types of software limitations. The purpose of this work is to describe our methodology for evaluating DECT registration accuracy and to apply this methodology to the Syngo algorithm using two physical phantoms that exhibit distinct types of motion: a deformable abdomen phantom to mimic deformation due to respiration and a rigid phantom to mimic the sliding motion that occurs between the chest wall and lungs due to respiration. Additionally, this work also quantifies the impact of registration errors on end‐product DECT images, that is, monoenergetic images and iodine images.

## 2 METHODS AND MATERIALS

### 2.A Image acquisition and processing

Both phantoms used in this study were imaged using a Siemens Healthineers SOMATOM Definition Edge scanner with the sequential scan technique, which is referred to as “Dual Spiral” by Siemens Healthineers. The following scan and reconstruction parameters were used for all phantom scans: low energy scan (80 kVp, 500 mAs, 0.5 s tube rotation time, 0.6 mm × 128 detector configuration, 2 mm slice thickness, 50 cm reconstructed field of view, 0.6 pitch, 9.3 mGy CTDI_vol_) followed by a high energy scan (140 kVp, 118 mAs, 0.5 s tube rotation time, 0.6 mm × 128 detector configuration, 2 mm slice thickness, 50 cm reconstructed field of view, 1.2 pitch, 12.2 mGy CTDI_vol_). To establish baseline values, phantom images were acquired without motion (static scan). For motion scans, the phantom was manipulated or deformed between the acquisition of low and high energy data.

The low and high energy images were exported to the Syngo software (version VB20) for postprocessing. The software automatically deforms the low energy image to the high energy image without any user interaction. End‐product images (mixed 120 kVp, monoenergetic, and iodine images) were then generated and exported to MIMvista (MIM Software, Inc, Cleveland, OH) to evaluate registration accuracy. The end‐product images are calculated using the deformable image registration and therefore represent the product of this deformation.

### 2.B Deformable abdomen phantom

**Fig. 1:**
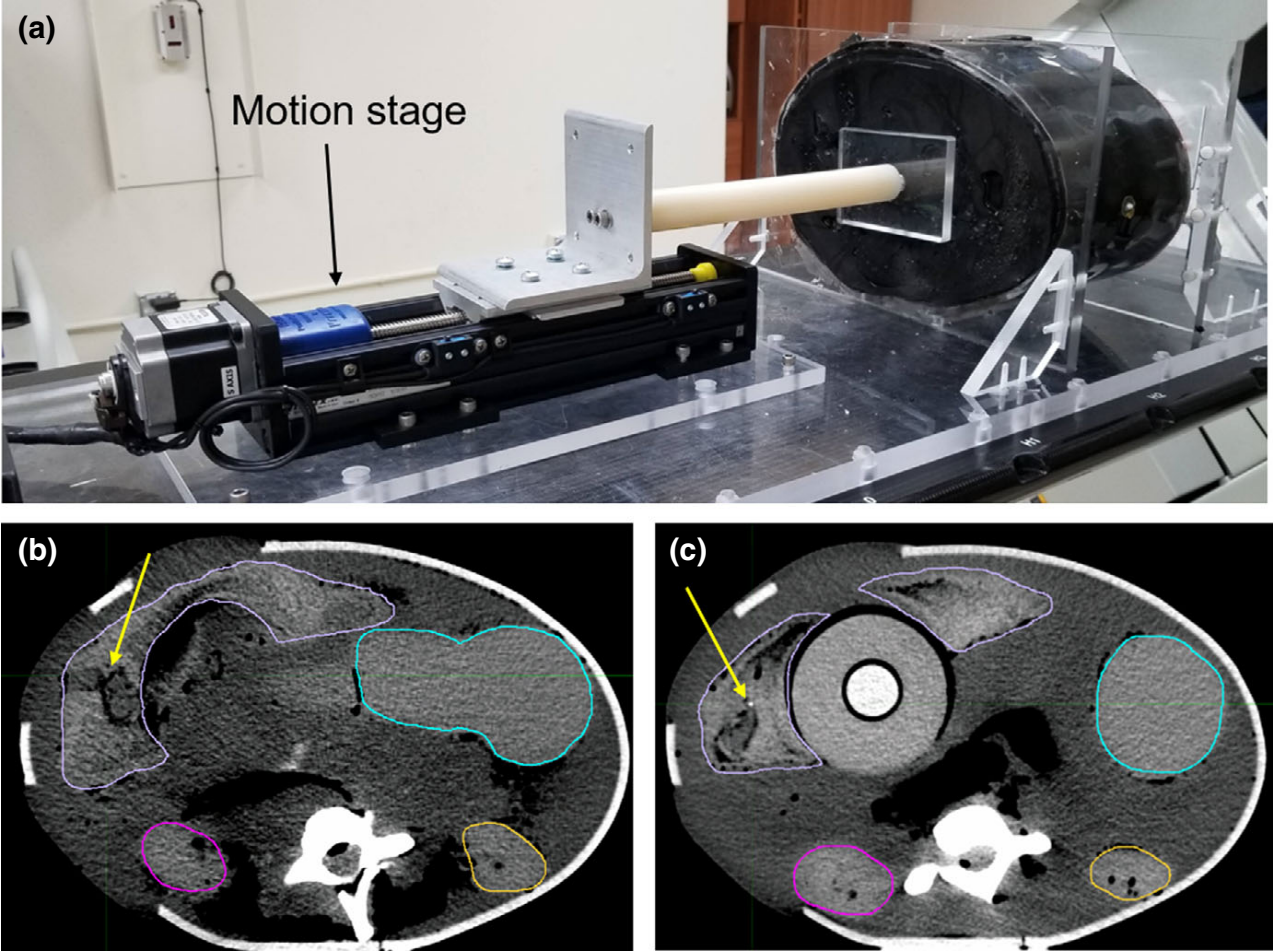
(a) PVC plastisol‐based deformable abdomen phantom with motion platform, (b) high energy 140 kVp image of the phantom with liver, kidneys, and stomach contoured, and (c) a different slice of the corresponding mixed 120 kVp image of the phantom with 20 mm programmed motion between scans. The arrow in (b) points to the “bifurcation #1” landmark, and the arrow in (c) points to the “liver fiducial” landmark referenced in Table 1.

To evaluate the accuracy of image registration for motion in the abdomen due to respiration, a PVC plastisol‐based deformable anthropomorphic phantom (approximately 20 cm A/P and 30 cm L/R) was used that contains objects mimicking the liver, kidneys, stomach, and vertebral bodies (Fig. 1).^12^ The phantom was deformed using a programmable motion stage with a piston attached to contact the inferior end of the phantom using a rectangular block of acrylic [Fig. 1(a)]. This motion stage is able to hold static positions or move dynamically; for this study, 20 mm of programmed static motion was applied to the phantom. Although this is external motion on the inferior end of the phantom and not the internal motion of abdominal organs, 20 mm of motion is consistent with the amount of motion observed for shallow breathing for various abdominal organs (pancreas, liver, kidneys, and diaphragm).^13^ In order to quantify iodine concentration accuracy reported by the Syngo software, 5 and 10 mg/mL iodine inserts (Gammex, Middleton, WI) were also imaged within this phantom. Registration accuracy was quantified using landmark analysis, contour agreement, and iodine concentration accuracy.

#### 2.B.1 Landmark analysis

Five landmarks were identified in the low energy image, the high energy image, and the mixed 120 kVp image (a linear combination of the deformed low and high energy images). The landmark points included the inferior aspect of the iodine insert (centroid of the bottom slice of the cylindrical insert), a distinct feature of one of the vertebral bodies (similar in appearance to a small bone spur), a marker in the liver [Fig. 1(c)], and two vessel bifurcations in the liver [“bifurcation #1” is shown in Fig. 1(b)]. The mean distance between these points was calculated for both the high vs low images and the high vs. mixed 120 kVp image, with the high energy image as the reference image in both cases. The mean distance or displacement between corresponding landmark points, called the target registration error, was calculated for these landmarks.^7^

#### 2.B.2 Contour agreement

The liver, right kidney, left kidney, stomach, and one of the vertebral bodies were contoured by a medical physicist on the high energy and mixed 120 kVp images for 20 mm of programmed motion [Figs. 1(b) and 1(c)]. The agreement between contours was quantified using the DSC, which is the ratio of two times the volume of the intersection of the two contours to the sum of the volume of the contours [Eq. (1)]. A DSC value of unity signifies perfect agreement between contours.

(1) $$DSC=\frac{2\left(A\cap B\right)}{A+B}$$

The DSC was calculated for the mixed 120 kVp image vs. the high energy image for 20 mm of programmed motion. However, since the high energy image has lower contrast than the mixed 120 kVp image, it is expected that there is some baseline level of disagreement between corresponding contours even if no motion is present. To quantify this baseline level of disagreement, the same comparison was performed for a pair of mixed 120 kVp and high energy images that were generated from a scan in which there was no motion or phantom deformation (static scan).

#### 2.B.3 Iodine concentration accuracy

The Liver VNC application in Syngo was used to generate iodine images with reported iodine concentration. The iodine concentration in the rod inserts was measured using the dual‐energy ROI tool in Syngo on a slice in the middle of the rod, the inferior end, and the superior end, and the average value was compared to the nominal concentration reported by the manufacturer.

### 2.C Sliding motion phantom

**Fig. 2:**
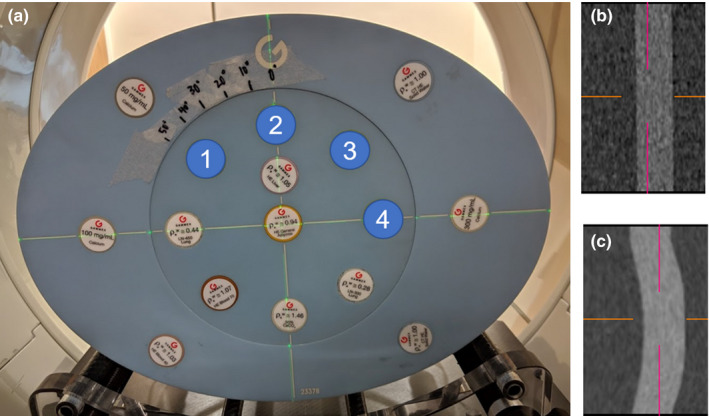
(a) Gammex Multi‐Energy computed tomography phantom used to simulate rigid sliding motion. (b) High energy and (c) deformed low energy image of the 5 mg/mL iodine rod imaged in the phantom with 15 degrees of rotation between high and low energy sequential scans. Labels 1, 2, 3, and 4 correspond to iodine inserts with 2, 5, 10, and 15 mg/mL of iodine, respectively

The Gammex Multi‐Energy CT phantom (MECT) was used to simulate the type of sliding motion that is encountered in the thorax between the expanding lungs and the chest wall. To mimic two organs sliding past one another, the inner portion of the MECT phantom was rotated while keeping the outer portion stationary (Fig. 2). Scans were acquired with inner phantom rotations ranging from 5° to 20°, which corresponds to an arc length of motion ranging from 0.9 to 3.5 cm. For context, AAPM's Task Group 76 on respiratory motion management summarized the magnitude of motion for various organs in the abdomen and found that the diaphragm can move as much as 3 cm in the superior–inferior direction for shallow breathing and as much as 10 cm for deep breathing.^13^ This phantom was imaged with a variety of tissue substitute rods and multi‐energy rods of various material concentrations. Iodine rods of various concentrations (2, 5, 10, and 15 mg/mL iodine) located within the inner portion of this phantom [Fig. 2(a)] were used to evaluate contour agreement and the impact of misregistrations on monoenergetic and iodine images. All inserts were cylindrical and thus there were limited point landmarks in the phantom.

#### 2.C.1 Contour agreement

Four iodine rods (2, 5, 10, and 15 mg/mL iodine concentration) imaged in the inner portion of the MECT phantom [Fig. 2(a)] were contoured on 50 keV monoenergetic images generated from static and rotated phantom scan acquisitions, and the DSC was calculated to evaluate contour agreement (monoenergetic image contours vs high energy image contours from the same scan acquisition).

#### 2.C.2 Monoenergetic HU and iodine concentration accuracy

To quantify monoenergetic HU accuracy for images acquired with phantom motion, the mean HU value was calculated for the contours of the iodine rods generated in the previous section and compared to the corresponding value for static images of the phantom. Additionally, using the Liver VNC application in Syngo, iodine images of the MECT phantom were generated, and the dual‐energy ROI tool was used to obtain the reported concentration for a slice in the middle of the rod, the inferior end, and the superior end. The mean reported concentration was then compared to the nominal concentration.

## 3 RESULTS

### 3.A Deformable abdomen phantom

Based on a visual qualitative assessment of the mixed 120 kVp images vs. high energy images of the abdomen phantom, it can be seen that the deformable algorithm performed well for the organs and the observed differences are mostly due to changes in air cavities in the phantom that are removed when the phantom is compressed. Some slight distortion was also observed in the vertebral bodies. Landmark analysis was performed to quantify registration accuracy, and Table 1 shows the results for five landmarks. Before deformable registration was performed, the distance between landmarks ranged from 5 to 14 mm when comparing the high vs. low energy images, which were acquired with 20 mm of programmed motion in between scans. The locations of landmarks were all within 1 mm when comparing the high energy images and the mixed 120 kVp images, which were created using deformable image registration. The mean target registration error based on this landmark analysis was 0.4 mm, indicating excellent registration accuracy.

**Table 1:** Distance between corresponding landmarks for images of the abdomen phantom with 20 mm programmed motion in the case of no registration (low vs. high energy images) and deformable image registration (mixed 120 kVp vs high energy images).

Landmark	Distance (mm)	
	Low vs high	Mixed vs high
Iodine insert	9.1	0.2
Bone	14.1	0.8
Bifurcation #1	5.8	0.6
Bifurcation #2	7.0	0.2
Liver fiducial	5.6	0.3
Mean registration error	8.3	0.4

Additionally, contour agreement, as quantified by the Dice similarity coefficient, was used to assess registration accuracy. Table 2 shows the Dice coefficients for five organs that were contoured based on mixed 120 kVp and high energy static images of the abdomen phantom, as well as images generated with motion. For the organs evaluated, the DSCs were similar for static vs. deformed images, indicating excellent registration accuracy.

**Table 2:** Dice similarity coefficients for various regions of interest for mixed 120 kVp vs high energy images of the abdomen phantom with no motion and 20 mm programmed motion.

Region of interest	Static	20 mm motion
Left kidney	0.933	0.938
Right kidney	0.914	0.914
Liver	0.941	0.939
Stomach	0.961	0.952
Vertebral body	0.957	0.950

Similarly, phantom deformations were not found to substantially impact iodine concentration accuracy with this phantom. For a 5.0 mg/mL iodine insert, the reported concentration in the Syngo software was 4.7 mg/mL for static images of the phantom and 4.5 mg/mL for 20 mm of programmed motion. For the 10.0 mg/mL insert, the reported concentration was 9.0 mg/mL for static images and 8.9 mg/mL for 20 mm of programmed motion.

### 3.B Sliding motion phantom

**Fig. 3:**
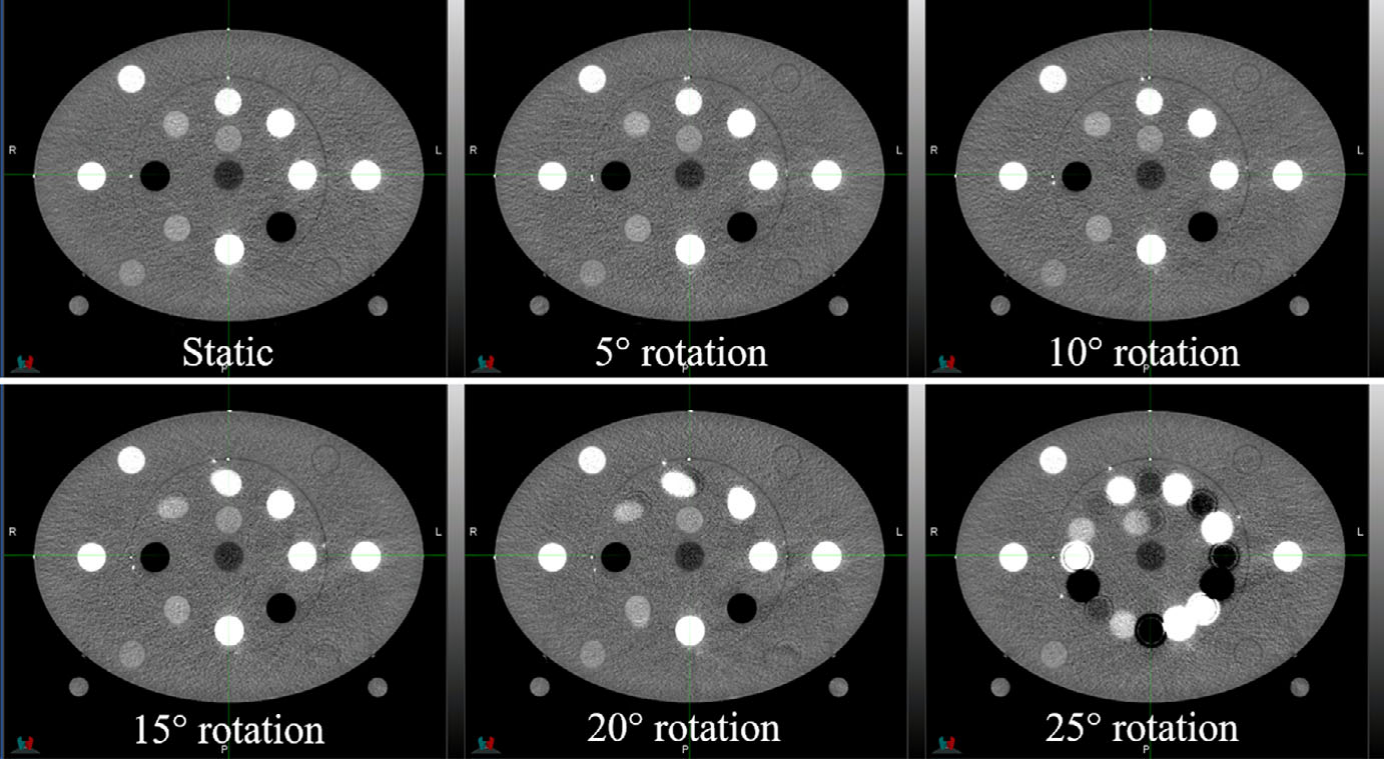
50 keV monoenergetic images generated from scan acquisitions of the multi‐energy computed tomography (MECT) phantom with various amounts of applied motion

For the Gammex MECT phantom, in which rotation of the inner portion of the phantom was used to mimic rigid sliding motion, Fig. 3 illustrates 50 keV monoenergetic images for varying amounts of motion. The registration algorithm had more difficulty with this type of motion than it did with the deformation introduced in the abdomen phantom. Registration errors were apparent in the resultant images and worsen with increasing motion magnitude. For 25 degrees of rotation, the registration algorithm broke down and was not able to correct for that large amount of motion (Fig. 3). The tissue substitute rods in the inner portion of the phantom were close to the motion interface and exhibit the most noticeable misregistration artifacts. Additionally, due to the regularization term in the algorithm, the rods in the phantom were bowed or warped along the superior/inferior axis in the deformed low energy image [Fig. 2(c)].

**Fig. 4:**
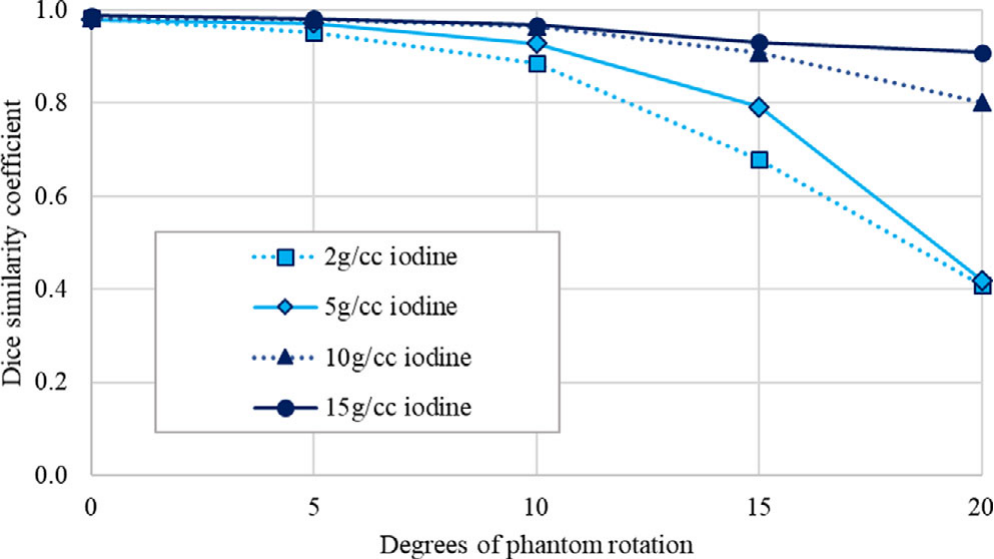
Dice similarity coefficient (140 kVp high energy vs 50 keV monoenergetic contours) as a function of phantom motion for various iodine rods imaged in the multi‐energy computed tomography phantom.

Contour agreement on 50 keV monoenergetic images was used to assess registration accuracy as a function of motion for the various iodine rods imaged in the inner portion of the phantom. Figure 4 shows that the DSC for these rods decreases for increasing phantom motion, indicating worsened registration accuracy for increasing amplitude of motion. This agrees with a qualitative assessment of the images in Fig. 3. The performance of the algorithm was generally worse for the lower iodine concentrations (2 and 5 mg/mL) due to the lower contrast of these rods in comparison to the phantom background material. For up to 10 degrees of phantom rotation, all rods have a Dice coefficient > 0.80, but for greater amounts of phantom rotation the Dice coefficient continues to decrease especially for the lower contrast rods. Of note, for 20 degrees of motion the 2 and 5 mg/mL iodine rods have a Dice coefficient of 0.41 and 0.42, respectively.

**Fig. 5:**
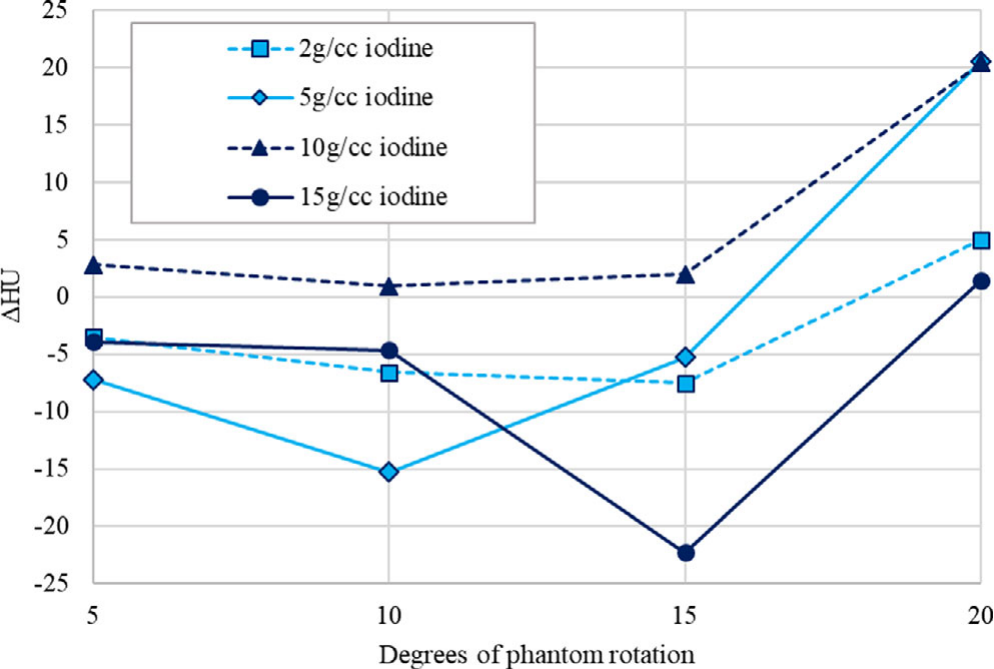
Change in HU with respect to static 50 keV monoenergetic images for various iodine concentration rods as a function of phantom motion in the multi‐energy computed tomography phantom.

In addition to contour agreement, changes in HU values in 50 keV monoenergetic images due to phantom motion were evaluated. Figure 5 shows changes in HU values, with respect to static images of the phantom, as a function of motion. All four of the rods had mean HU values within 25 HU of the values obtained from static images, for all magnitudes of motion investigated, demonstrating that the registration errors had little effect on the mean HU values in the iodine rods.

**Fig. 6:**
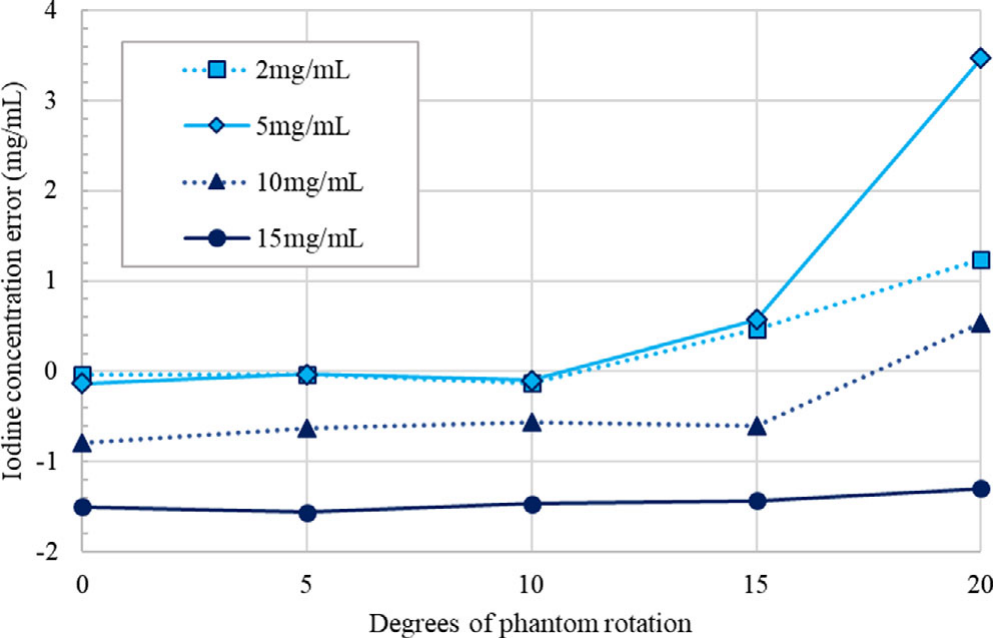
Iodine concentration error in the center of iodine rods of various concentrations as a function of phantom motion for rigid sliding motion in the multi‐energy computed tomography phantom.

Iodine concentration accuracy was also evaluated as a function of phantom motion (Fig. 6). In general, the concentration in the central portion of the iodine rods was fairly consistent between motion images and static images for smaller amounts of motion (≤10 degrees of motion). For larger amounts of motion, the concentration accuracy was degraded. Additionally, large local concentration errors > 3mg/mL were observed in areas of misregistration.

## 4 DISCUSSION

This study evaluated the accuracy of the Siemens Healthineers Syngo deformable image registration algorithm used for dual‐energy CT postprocessing to account for patient motion. Notably, this algorithm is used for all DECT applications available in the software including bone removal, liver virtual noncontrast imaging, and generation of monoenergetic images. Because of the broad application of this algorithm for sequential scan and split‐filter DECT acquisitions, we chose to test the algorithm using two distinct types of motion, deformation due to respiration in the abdomen and rigid sliding motion as observed in the thorax. The validation testing and metrics recommended by AAPM’s TG‐132 were incorporated into our study.

**Fig. 7:**
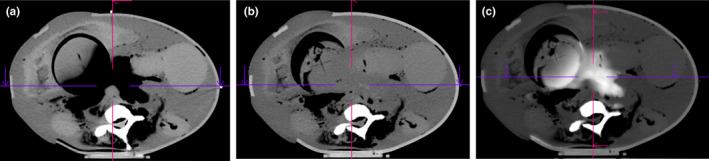
(a) 80 kVp low energy deformed image, (b) 140 kVp high energy image, and (c) resulting 120 keV monoenergetic image illustrating hyperintense artifacts caused by the algorithm's inability to handle compressed gas.

For motion in the abdomen, we used a deformable, anthropomorphic abdomen phantom and found that the registration algorithm performed very well. Landmark analysis resulted in a target registration error of <1 mm, and contour analysis of end‐product dual‐energy images resulted in DSCs > 0.90. Both quantitative results indicate excellent registration accuracy and meet the tolerance levels recommended by TG‐132. Additionally, for the two iodine rods imaged in this phantom, the concentration reported for motion images was within 0.2 mg/mL of values for static images. It should be noted, however, that the Syngo algorithm is volume‐preserving and includes regularization to prevent discontinuities in the deformation field. Therefore, one limitation of the algorithm observed in this study was its ability to handle changes in volume, as would be encountered with compressed air (e.g., bowel gas). When motion was applied to the inferior end of our deformable anthropomorphic abdomen phantom, air pockets were compressed which was not fully accounted for with the deformable registration algorithm. Although it did not affect the visualization of organs in the mixed 120 kVp images, it did cause hyperintense artifacts in monoenergetic images as illustrated in Fig. 7. Clinically we have observed similar artifacts due to changes in bowel gas for split‐filter scans of the abdomen.

For rigid sliding motion, evaluated by rotating the inner portion of the Gammex MECT phantom with respect to the outer portion of the phantom, the registration algorithm was less successful and produced clearly visible registration errors for larger amounts of motion (Fig. 3). This registration error resulted in worsened contour agreement (Fig. 4) and errors in iodine concentration (Fig. 6). For clinical imaging, imperfect registration can result in hyperintense artifacts in dual‐energy images that can obscure relevant anatomy and lead to an overestimation of iodine concentration. Clinically, our institution has observed warping of bony anatomy due to inappropriate application of deformable image registration to rigid structures. These errors could be minimized with the ability to perform a rigid registration rather than a deformable one; however, this is not possible within the software at the time of this publication.

Due to restrictions within the current software, limitations of this study are that the full suite of testing recommended by TG‐132 could not be performed and only the end‐product images (mixed 120 kVp, monoenergetic, and iodine images) were evaluated rather than the actual deformed low energy images. The Siemens Healthineers Syngo software does not allow the user to export the deformed low energy image, nor does it allow the user to view or export the deformation vector field. Additionally, there are no analysis tools within the software, aside from image blending, to aid the user in performing evaluation and QA of the image registration. Therefore, it was not possible to use digital phantoms with known deformations for testing, as recommended by TG‐132. However, given the current software limitations, this work proposes a methodology to quantitatively evaluate registration accuracy that can be used by dual‐energy CT users of various commercial platforms. Although our work uses a custom deformable abdomen phantom, the Gammex MECT phantom is commercially available and users can adapt this methodology to phantoms available in their clinic.

Another limitation is that our study focuses on the sequential scan technique for dual‐energy CT acquisition. This was done in order to facilitate phantom motion and deformation in a controlled manner between the acquisition of low and high energy scans. In the case of the Syngo software, the same deformable registration algorithm is applied to all dual‐energy acquisition techniques, including sequential scan and split‐filter imaging. Therefore, our methodology can be used to broadly test the image registration algorithm even when it is applied to dual‐energy techniques (e.g, split‐filter and dual‐source) in which motion is less of a concern due to the smaller time interval between the acquisition of high and low energy data. However, based on our clinical experience, motion can still be an issue for these other scan techniques, as we have observed artifacts due to bowel gas motion for split‐filter pancreas studies. An additional limitation of this study is that unlike the MECT phantom, a range of motion magnitudes was not investigated for the deformable abdomen phantom. However, 20 mm of programmed motion at the inferior aspect of the abdomen phantom did result in a range of deflections within the deformable phantom (5–14 mm based on our landmark analysis). Another limitation of this study is that sliding motion was created by rotating the inner portion of the Gammex MECT phantom; it is possible that the deformable registration algorithm may have handled sliding motion created by translation differently.

This study highlights the importance of understanding the image registration algorithms implemented in commercial dual‐energy postprocessing software, especially given the increasing use of dual‐energy CT for different anatomical sites and clinical applications. In the case of Syngo, the algorithm was successful for abdominal deformations but was less successful for rigid sliding motion. Testing different types of motion is important to understand the limitations of the algorithm and how those limitations may impact different types of DECT images.

## 5 CONCLUSIONS

This work proposes the first method to evaluate a deformable registration algorithm used in dual‐energy CT postprocessing following the recommendations of TG‐132. Based on the results of our study, the Siemens Healthineers Syngo deformable image registration algorithm performed very well for deformations < 2 cm in the abdomen and can be used clinically for abdominal sites with limited motion (e.g., pancreas and liver). For rigid sliding motion, as would be encountered in the thorax due to the lungs expanding and sliding along the chest wall and spine, the algorithm resulted in visible registration errors and iodine concentration errors. Thus, it is not recommended to use the algorithm in areas that exhibit rigid sliding motion as it can result in artifacts and distortion in the resulting images and quantitative errors in dual‐energy CT images.
